# Polymeric Biodegradable Stent Insertion in the Esophagus

**DOI:** 10.3390/polym8050158

**Published:** 2016-04-26

**Authors:** Kai Yang, Christopher Ling, Tianwen Yuan, Yueqi Zhu, Yingsheng Cheng, Wenguo Cui

**Affiliations:** 1 Department of Radiology, Shanghai Jiao Tong University Affiliated Sixth People’s Hospital, Shanghai Jiao Tong University, 600 Yi Shan Road, Shanghai 200233, China; 98211yangkai@163.com (K.Y.); ytw806@163.com (T.Y.); zhuyueqi@hotmail.com (Y.Z.); 2 Department of Orthopedics, The First Affiliated Hospital of Soochow University, Orthopedic Institute, Soochow University, 708 Renmin Road, Suzhou 215006, China; cwfling@uwaterloo.ca; 3 Nanotechnology Engineering, University of Waterloo, 200 University Ave W, Waterloo, ON N2L 3G1, Canada

**Keywords:** biodegradable stents, polymer, dysphagia, esophageal perforation/leak, malignant esophageal stricture, (refractory) benign esophageal stricture

## Abstract

Esophageal stent insertion has been used as a well-accepted and effective alternative to manage and improve the quality of life for patients diagnosed with esophageal diseases and disorders. Current stents are either permanent or temporary and are fabricated from either metal or plastic. The partially covered self-expanding metal stent (SEMS) has a firm anchoring effect and prevent stent migration, however, the hyperplastic tissue reaction cause stent restenosis and make it difficult to remove. A fully covered SEMS and self-expanding plastic stent (SEPS) reduced reactive hyperplasia but has a high migration rate. The main advantage that polymeric biodegradable stents (BDSs) have over metal or plastic stents is that removal is not require and reduce the need for repeated stent insertion. But the slightly lower radial force of BDS may be its main shortcoming and a post-implant problem. Thus, strengthening support of BDS is a content of the research in the future. BDSs are often temporarily effective in esophageal stricture to relieve dysphagia. In the future, it can be expect that biodegradable drug-eluting stents (DES) will be available to treat benign esophageal stricture, perforations or leaks with additional use as palliative modalities for treating malignant esophageal stricture, as the bridge to surgery or to maintain luminal patency during neoadjuvant chemoradiation.

## 1. Introduction

Esophageal strictures (ES) are commonly caused by benign and malignant diseases, and according to the method can be divided into operative and non-operative treatment. Nonoperative causes include esophageal reflux, external beam radiation, esophageal sclerotherapy, caustic ingestions, and advanced cancer. Operative causes include surgical anastomosis, after endoscopic submucosal dissection (ESD), or endoscopic mucosal resection (EMR) for early esophageal neoplasms. Dysphagia, malnutrition, weight loss, aspiration and respiratory failure caused by benign or malignant esophageal strictures are frequently encountered problems with ES [1]. Esophageal strictures are difficult to manage conservatively and they usually require balloon dilation and/or stent insertion, which play a vital role in alleviating obstructive symptoms (dysphagia) or treating the stricture-related complications and improving the quality of life for the patient.

The use of stents for esophageal strictures have evolved rapidly over the past 30 years from rigid plastic tubes to flexible self-expanding metal stents (SEMS) and self-expanding plastic stents (SEPS). According to the implantation time, SEMS can be divided into two categories: permanent stents and retrievable temporary stents. The design of SEMS can be divided into two classes: partially covered metal stents and fully covered metal stents. Current SEMS and SEPS insertion have become an optimal and effective alternative to the treatment of malignant and benign esophageal diseases. However, the use of stents is associated with several common problems including migration, tissue ingrowth, restenosis and repetitive procedures.

**Figure 1:**
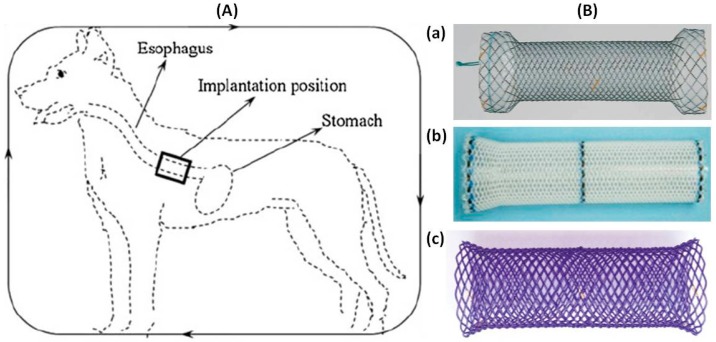
Stent insertion (**A**) [4] and stents (**B**) currently used for benign or malignant esophageal strictures: (**a**) Niti-S covered stent (Taewoong Medical); (**b**) Polyflex stent (Boston Scientific); and (**c**) ELLA-BD stent (ELLA-CS). Copyright permission from Springer.

The past two decades, the development of medical application of polymeric biocompatible and biodegradable materials have made significant progress. The polymeric biodegradable stents (BDSs) fabricated from these biocompatible and biodegradable materials have been developed to overcome some shortcomings encountered with SEMS or SEPS [1]. The cardiovascular stent market is currently the dominant driving force for the research and development of BDSs [2]. Limited symptomatic relief and a high rate of adverse incidents encountered with SEMS or SEPS has led to the use of BDSs since 1997 [3]. Since BDSs do not require endoscopic removal and alleviate dysphagia similarly to SEMS or SEPS, they are beginning to be used in the treatment of benign or malignant esophageal strictures. BDSs or biodegradable drug-eluting stents (DES) insertion as a “bridge to surgery” or palliative treatment can improve symptoms and allow enteral nutrition until staging or neo-adjuvant treatment is completed (Figure 1).

SEMS and SEPS stent placement is a commonly used, minimally invasive method to treat benign or malignant esophageal strictures. Due to the limited success of SEMS and SEPS for treating esophageal strictures, such as bleeding, esophageal fistula, migration, retrosternal pain, tissue ingrowth, restenosis and repetitive procedures. Therefore, considerable effort should be research to avoiding those complications in patients. The characteristic features of BDSs, such as solubility and natural absorption over a period time, be able to prevent those complications reported for the use of SEMS, the BDSs have provided an additional therapeutic option. However, BDSs are much less known in clinical utility and experience. This review focuses on the current experience with polymeric BDSs for benign and malignant esophageal diseases.

## 2. Polymeric Biomaterials

A polymeric biomaterial is a non-biological material used in a medical device and application and designed to interact with biological systems. A polymeric biomaterial [5,6,7,8,9,10,11,12,13,14,15] can be: (1) inert, does not trigger any reaction in the host; (2) bioactive, ensures a stable performance for long durations or the period desired; and (3) biodegradable, it can be resolved through natural chemical degradation or effectors such as bacteria. The main characteristic of these materials for medical application are the absence of carcinogenicity, immunogenicity, teratogenicity and toxicity.

Commonly used biomaterials include magnesium based alloys and synthetic polymers, which are mainly α-hydroxy acids [5,6,7,8,9,10,11,12,13,14,15] such as: polylactic acid (PLA), poly-l/d-lactic acid (PLLA/PDLA), polyglycolic acid (PGA), polycaprolactone (PCL), polydioxanone (PDX), polyhydroxylbutyratevalerate (PHBV), polyglycolic acid/polylactic acid (PGLA), polyacetyglutamic acid (PAGA), polyorthoesters (POE), polyethylene oxide/polybutylene terephthalate (PEO/PBTP), hyperbranched polyphosphates (HBPPs) [7], styrene-*b*-isobutylene-*b*-styrene (SIBS) [8], poly(acrylic acid)–poly(ethylene glycol) (PAA-PEG) [9], and poly(lactide-ε-caprolactone) (PLCLs) [10]. PGA and PDX are relatively fast-bioabsorbing materials (weeks to months), on the contrary PLA and PCL are relatively slow-bioabsorbing materials (months to years). In general, polymers will degrade at a slower rate than magnesium alloys.

The ideal polymer should: (1) be sufficiently structurally stable and posses high mechanical properties until the completion of the therapeutic purposes; (2) not invoke inflammatory or toxic responses; (3) be able to be completely metabolized in the body after achieving its purpose; (4) be easily processable into the desired form; (5) demonstrate acceptable shelf life; and (6) be easily sterilized.

The major advantages of synthetic polymers are: (1) good biocompatibility; (2) adjustable composition and physical-mechanical properties; (3) low coefficients of friction; (4) easy processing and workability; (5) ability to change surface chemically and physically; and (6) ability to immobilize cells or biomolecules inside or on the surface.

According to these features, BDSs can be made from different synthetic polymers (PLA and PGA) or their copolymers (PDX). Degradation of the BDSs are hydrolytic, the speed of biodegradation is dependent not only on size and structure of polymer, but also influenced by surrounding environment, such as temperature, pH and type of body tissue/fluid [5,6,7,8,9,10,11,12,13,14,15].

## 3. Prerequisites for Polymeric Biodegradable Stents

Stent is a cylindrical medical equipment to expand a stricture lumen in order to maintain the patency of the lumen. When selecting a polymer for the BDS, there are several conditions need to consider, such as the strength to avoid potential immediate recoil, the rate of degradation, lack of toxicity, and biocompatibility of the polymer and the degraded products with the target organ pipe wall, vessel or digestive tract. The change of mechanical properties and drugs release of BDSs would directly depend on the rate of stent degradation, which can be controlled through the choice of polymer, passivation agents and stent manufacturing process. Studies presented by Freudenberg *et al.* [5] and Gunatillake *et al.* [6] researched the technical feasibility of BDSs including factors such as exterior design, stent forming, coating modification and drug loading technology.

The present invention of BDSs [5,6,7,8,9,10,11,12,13,14,15] include a method of designing and manufacturing an improved braided BDS which is different from conventional practices used to make a braided metal wire stent. The method involves selecting a specific biodegradable polymer based on a desired stent functional degradable time and stent radial force. Then, according to the different requirements of the organs, the degradable polymer materials are select to meet the requirements (degradable time and stent radial force).

An ideal BDS should have the following advantages: (1) a mechanical performance comparable to metal stents; (2) complete biodegradability after a certain period of mechanical support with target organ pipe wall and non-toxic side effects of degradation products; (3) good compliance to make it easier to arrive at the lesion and penetrate the lesion lumen; (4) good histocompatibility; (5) a drug loading capacity better than metal stents and a slow local delivery; and (6) the ability to be tracked.

## 4. Advantages of Polymeric Biodegradable Stents

**Figure 2:**
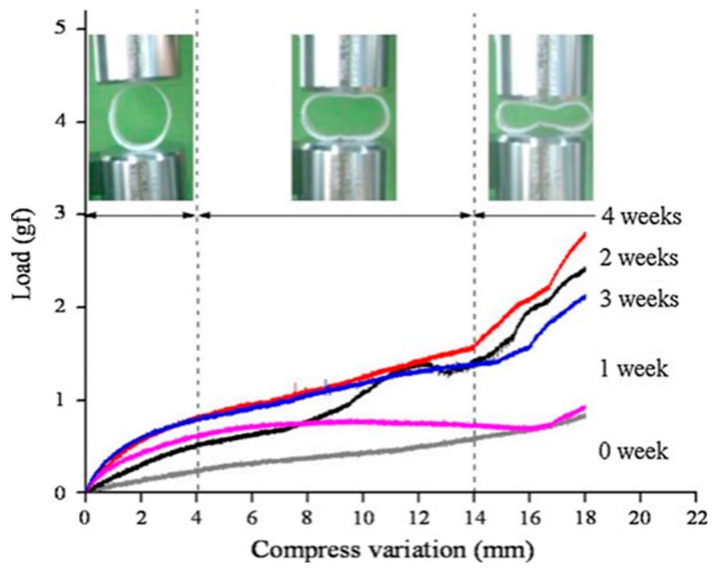
Influence of *in vitro* degradation time on anti-compressproperty of PCLA ring and inserted photos showing the process of the compression [4]. The test shown that a gradual increase in compressive strength and differed from previous reports that the biodegradation process of polymer led to a decline inmechanical properties. Copyright permission from Springer.

There are several advantages of using polymeric BDSs over metallic stents [16]. One of the largest advantages is the BDS can naturally decompose into non-toxic chemical species over time. In addition, BDSs may be manufactured at a relatively low cost, because the vacuum heat treatment and chemical cleaning is not required. BDSs have the ability to carry and release drugs, whereas metal stents are not able to do [16]. The BDSs can release the drugs during the scaffold degradation process resulting in tunable release profiles by changing the degradation rate of the stent via adjusting the composition of the BDS. In addition, varying the polymer composition on the abluminal and luminal sides may yield many more benefits that metal stents offer. These benefits include facilitating a more targeted drug delivery and inducing different cell growths on different sides, encouraging endothelialization on the luminal side at the same time as limiting smooth muscle cell proliferation on the abluminal side [17]. Avoid repeat percutaneous revascularization or surgical intervention, which would be used in metallic stents if required subsequently [18]. Avoiding metallic stents may also prevent the jailing of side branches and difficulties with overhang at ostial lesions. SEMS would fully expand within 24 h of implantation which can potentially limit the late favorable positive remodeling and occur the development of new lesions, whereas BDSs do not have this shortcoming [4,19] (Figure 2). The magnetic resonance imaging (MRI) or computer tomography (CT) compatibility of BDSs may lead to a better diagnostic because there is no blooming from metallic artifact [20]. In addition, BDSs may allay patient concerns over permanent implant; BDSs can disappear over a period of time meaning that they may reduce the long-term risks of stent thrombosis and need long-term dual antiplatelet therapy [21]. The ideal esophageal stents would be easily local, resistant to migration, resulting minimal tissue response, prevent tissue ingrowth, easy retrieval, and patients with good tolerability without discomfort or nausea.

## 5. History of Polymeric Biodegradable Stent Development

In 1988, Stack *et al.* [22] fabricated the first fully BDS, which was fabricated from knitted PLLA at the Duke medical center in USA. The stent could withstand up to 1000 mmHg in the extrusion, making its radial strength within one month and be completely degraded in nine months. Polymers have been widely used in DESs, primarily as delivery vehicles for drug coatings in 1991 [23,24]. The polymers suggested for BDSs are PLLA, PDLA, PGA and PCL. Those polymers were designed as not only a self-expanding but also balloon expandable stent. Another proposed design is a combines polymeric with a metallic backbone to increase strength and prevent recoil. In 1996, Ye *et al.* [25] used a PLLA/PCL blend stent impregnated with a recombinant adenovirus carrying the β-Gal reporter gene and demonstrated a successful transfer and expression of this gene in cells of the arterial wall in rabbits. In 1997, PLLA was associated with an intense inflammatory reaction, whereas a minimal inflammatory reaction was observed with molecular mass >300 kDa [26]. In 1998, Igaki and Tamai [27] further refined the design to PLLA monofilaments in a zigzag helical coil configuration with 0.17mm thick struts. This arrangement resulted in a reduced vascular injury at the implantation site, reduced initial thrombus deposition and cut down neointimal proliferation. Another interesting concept is the design of multi-layered biodegradable stent by Eury *et al.* [28], which is made from variety of polymers such as PLLA, PGA, PCL, poly-orthoesters or poly-anhydrides. The unique function of this stent is one layer to solve the structural requirements of the stent and other layer control the drug release. The stacked structure allows multiple loading of different materials with different drugs all within a single stent.Through an appropriate configuration of the layers drug release characteristics can be adjusted. Yamawaki *et al.* [29] used a high-molecular-weight PLLA Igaki-Tamai stent loaded with ST638 (Tranilast, a specific tyrosine kinase inhibitor) or ST494 (an inactive metabolite of ST638). It showed that the extent of neointimal formation and geometric remodeling were significantly less at the ST638 loaded stent site then at the ST494. In 1999, Igaki and Tamai [30] compared the improved Igaki-Tamai stent to Palmaz-Schatz stent, it shown that there no stent thrombosis and no significant differences in minimal lumen diameter (MLD) at six months. Histological examination revealed no inflammation and minimal neointimal hyperplasia on PLLA stent struts. Hietala *et al.* [31] conducted a 34-month study in rabbit model using a stent made of copolymer l- and d-lactide (l/d ratio 96%/4%) in 2001. This is the longest known research study using polymer stent and first reported complete endothelialization at 3 months with no inflammatory response after 6 months.Stent hydrolysis was evident at 12 months and completely disintegrated in 24 months. Stent was gradually has being replaced by fibrosis while maintaining patency of the lumen at all time.

In 2004, Vogt *et al.* [32] used a PLA eluting poly(d,l)-lactic acid (PDLLA) and reported a slow release profile of PLA with an exponential function. Starting with a daily release from 5 to 8 μg, decrease to 1 μg at 4 weeks and stopping completely at 3 months. Overall, the stent demonstrated mechanical stability during the entire duration. The histomorphometric analysis at three weeks demonstrated inhibition of neointima formation by 53% and 44% with the PLA-loaded PDLLA when compared to the normal PDLLA stent and metal stent. This mean the PLA-load increase the durability of the stent over 3 months.

## 6. Preclinical Studies with Polymeric Biodegradable Stents

**Figure 3:**
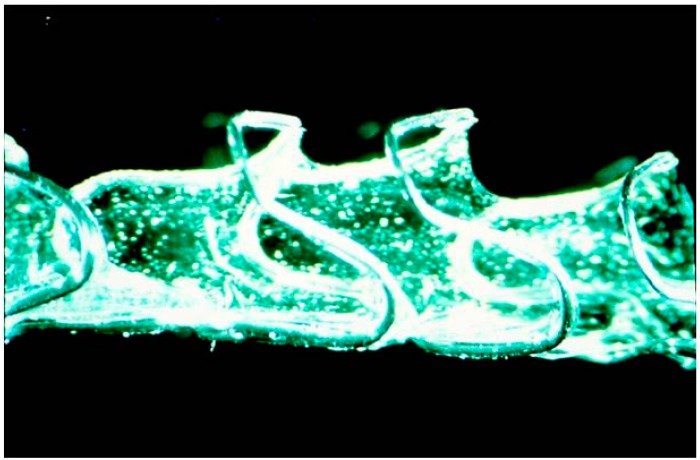
Polymer test strip cast asymmetrically on the coil stent vehicle [33]. The polymer was processed to strips 75 to 125 μm in thickness of specimen.The strips were cast longitudinally onto a balloon-expandable stent and covered ≈ 90° of the stent circumference. Copyright permisson from Wolters Kluwer Health Inc.

The first PLLA-BDS [22] shown minimal thrombosis, moderate neointimal growth, and a limited inflammatory response in porcine coronaries. Initial experimental studies with biodegradable polymers, which included PLLA, PDLA, PCL, poly-hydroxybutyrate-hydroxyvalerate, and poly-orthoester coatings as films on the circumferential surface of coil wire stents in the porcine coronary arteries, were disappointing. After 30 days, the histopathology revealed that a significant inflammatory reaction and endometrlosis with extensive cell infiltration of these coatings. There was also evidence of medial necrosis and pseudoaneurysm formation. Due to difficultly in manufacturing compatible biodegradable materials with the capability of limiting inflammation and restenosis, BDSs were not developed on the same scale as bare metal stents [33] (Figure 3). The loss of radial strength over time is another problem that increase the risk of fracture and migration of stent [34,35,36]. Furthermore, the biological compatibility of the long-term biological degradation products was relatively unknown and focused on absorbent of biodegradable polymers in coronary arteries [32]. The BDSs absorbing water led to chronic swelling, which has been shown to influence the degree of hyperplasia of the endometrium [37].

## 7. Preclinical Studies with Polymeric Biodegradable Esophageal Stents

The first report was published in 1993 [38] in a rabbit experimental model of urethral stenosis treated with a biodegradable self-reinforced stent made of PLLA. The polymeric BDSs in the esophagus under study are made from PDX, a monocrystalline polymer, with a 55% crystalline structure [39]. In living tissue, hydrolytic breakdown the crystalline structure into smaller fragments of low molecular weight products and then PDX being degraded. PDX has a stronger resistance to hydrolytic compared to PGA or PLC, which with faster degradation times of the stents. It is the longer persistence of the PDX that allow adequate time for esophageal reconstruction. The integrity and radial expansion force of stent can be maintained at 6–8 weeks and disintegrates in 11–12 weeks following the implantation [39]. The initial value of radial force being maintain in the first five weeks in physiological saline solution (pH 7, 37 °C). After seven weeks, the radial force was about two-thirds of the initial force and after 9 weeks about half of the initial force. After 2–4 months, the stent was completely degraded. The stent degraded faster with a lower pH, so it is advised to prescribe proton-pump inhibitors to prolong stent integrity. These new stents achieve constant radial force that similar to metallic stent, because of the advantage the stent do not need be removed. Until now, PDX-BDSs could be an option for benign refractory strictures in the gastrointestinal tract.

**Figure 4:**
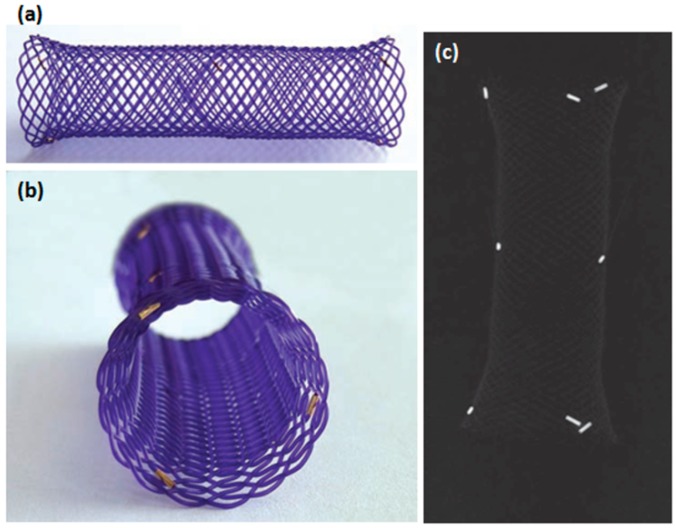
The EllA BD stent (Ella-SX, s.r.o., Hradec Kralove, Czech Republic) is uncovered and made of polydioxanone (**a**,**b**). The EllA BD stent has radiopaque markers on both ends and in the center (**c**) [43]. It is the markers that the stent is radiotransparent and it letsstents release convenient and visual. Copyright permisson from Taylor & Francis.

In 2010, Battersby *et al.* [40] used a biodegradable PDX self-expanding stent for treatment of benign esophageal stricture in a cat. Four months later, an examination indicated the BDS was no longer present and the esophagus showed no signs of obstruction. Yu *et al.* [4] designed a series of tests with new BDSs for the treatment of esophageal stenosis in dog models to investigate its properties. Some of which include shape memory effects, compression properties and the influence of *in vitro* degradation of the polymer matrix on its shape recovery and dilation force. The stent manufactured from poly (ε-caprolactone-*co*-dl-lactide, weight ratio of 1/9) (PCLA) copolymer. The deformed stent needed approximately 36 s to recover its initial shape *in vitro* in 37 °C warm water. The primary *in vivo* animal experiment revealed that the stent deform could be triggered by body temperature and expected to become a nearly-cylinder to support esophageal wall. Hence, the biodegradable polymer stent has giant potential to replace conventional metallic stents for treatment esophageal stenosis. Pauli *et al.* [41] evaluated the ability of ELLA-BDS (ELLA-CS, Hradec Kralove, Czech Republic) to reduce the aggressive stricture formation that occurs in a porcine model of circumferential endoscopic esophageal mucosal-resection (EEM) (Figure 4). The BDS showed little reduction in diameter or proximal dilation for the first six weeks. The survival time of the BDS was significantly longer than in the control group (9.2 weeks *vs.* 2.4 weeks).The timing of stricture formation correlated with the loss radial force and stent disintegration. A retrospective review of the records for dogs (*n* = 6) with RBES [42] found that all dogs had short-term improved dysphagia after BDS implantation. Complications included regurgitation (4/6), recurrence stricture (3/6), and stent migration (3/6). Three dogs required intervention due to these complications. The stent is radiotransparent, has radiopaque markers on both ends and in the center [43] (Figure 4).

## 8. Clinical Studies with Biodegradable Esophageal Stents

### 8.1. Benign Stricture

PLC-BDSs have been developed for treating patients with benign stenosis [44,45,46]. BDSs are a promising therapeutic option and were originally developed to manage esophageal strictures because it does not require removal and overcome some of the drawbacks of SEMS or SEPS. When migrated, gastric acid can dissolve and accelerate hydrolysis of stent, which avoid further injure and potential morbidity [44].

The first experience with a PLLA-BDS (InStent, Eden, MN, USA) for the treatment of benign esophageal strictures was reported by Goldin *et al.* [47] in 1996. The characteristics of PLLA are expansion, providing radial force and biodegradable in 3–6 months. This coil stent is wrapped tightly onto a catheter, with a thin wire holding its proximal and distal ends to a diameter of 10 mm. After endoscopic placement, the stent was released and expanded spontaneously to its design diameter of 14 or 16 mm (length of 6–10 mm) in a few minutes. In three patients, the BDSs collapsed within 3 weeks and caused recurrent dysphagia. In two patients, with an improved stent design and patency of stent was improved. PLLA-BDS (EsophaCoil; InStent, Eden Prairie, MN, USA) was first used in USA, for benign esophageal stricture due to radiation injury, by Fry and Fleischer [3] in 1997. The radial force of BDSs is weaker than that of conventional metal stents, so additional expansion using balloon dilatation is require after stent insertion.

**Figure 5:**
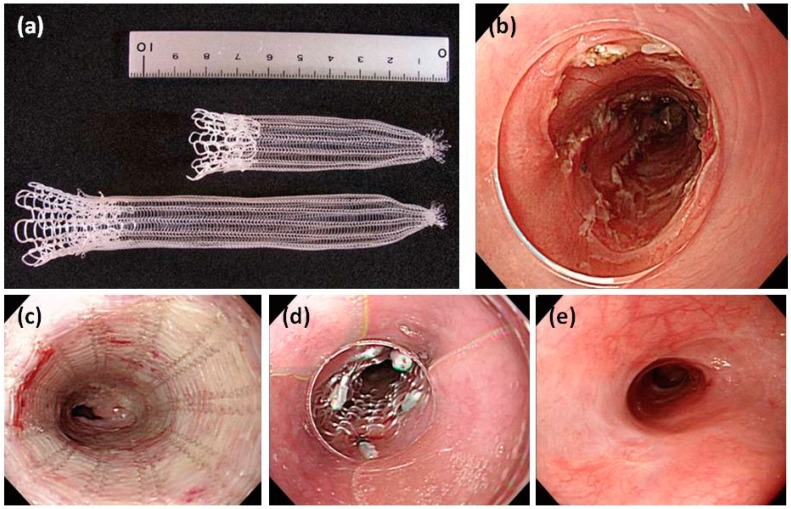
The PLLA esophageal stent (Tanaka–Marui stent; Marui Textile Machinery Co., Ltd., Osaka, Japan) (**a**); the mucosal defect after ESD (**b**); the released PLLA stent (**c**); fixation of the rostral side by endoscopic clips (**d**); and the view at six-month follow-up (**e**) [49]. Copyright permission from Baishideng Publishing Group Inc.

At the time, biodegradable stents were still in the investigative stage until ten years later, BDSs have been developed. In 2006, Tanaka *et al.* [48] reported modified stents were made of PLLA, which have sufficient radial force similar to those clinically applied esophageal stents and the PLLA-BDS was named Tanaka-Marui stent. Saito *et al.* [49,50] reported results from two series of patients who received the Tanaka-Marui stents (Marui Textile Machinery, Osaka, Japan) (Figure 5). The largest cohort consisted of 13 patients (six BES, two caustic and four anastomotic, and seven RBES, seven esophageal cancers following endoscopic mucosal dissection), which shown that the Tanaka-Marui stent underwent spontaneous migration in 10/13 patients (77%) within 10–21 days after insertion. Clinical success (complete relief of dysphagia) was observed in all cases within the follow-up period of 7–24 months. Follow-up studies have shown the PLLA-BD stent had a low stent-related complication rate. The history of stent degradation within the esophagus and the tolerability of the degradation process over a period of time were not sufficiency assessed since a high early stent migration rate was observed in the three studies.

**Figure 6:**
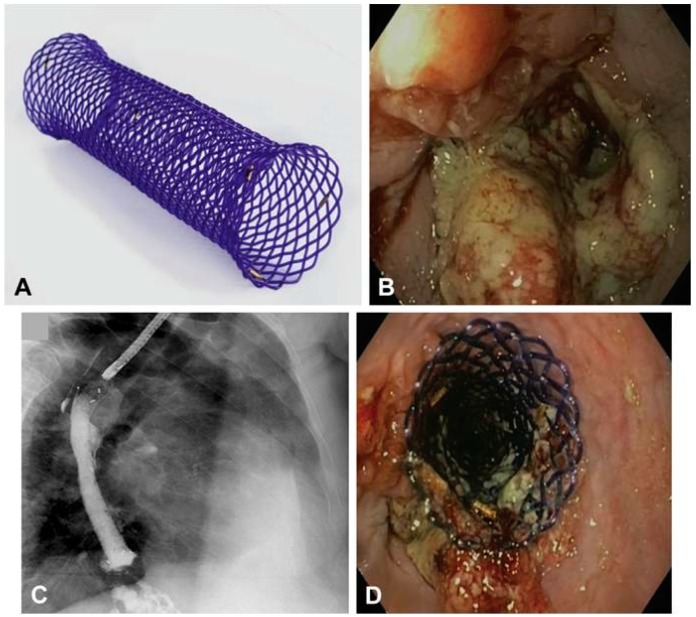
(**A**) Stent; obstructing adenocarcinoma in the distal esophagus (**B**); fluoroscopic control of stent deployment with radiopaque contrast agent (**C**); and endoscopic control of Ella-SX BD stent position (**D**) [75]. Preliminary clinical application show that the stent relieve obstruction treatment effect is obvious. Copyright permission from Elsevier.

In 2009, another novel stent (EllA esophageal stent, EllA-SX, s.r.o., Hradec Kralove, Czech Republic) [38] composed of the biodegradable polymer PDX was used (and currently the only one used clinically). The stent is currently available in four diameters (stent body of 18, 20, 23 and 25 mm) with lengths ranging from 60 to 135 mm and assembled onto a 9.4mm (28 F) delivery system. After release, the stent gradually expands, achieving its preformed diameter after 24–48 h. The stent gradually degrades by random hydrolysis of its molecular ester bonds. Integrity and radial force are maintained completely for approximately six weeks following implantation. From seven to nine weeks, it had two-thirds of the initial integrity and radial, after the nine weeks it had one-third of the initial integrity and radial, and the average time for complete degradation of the stent is reported to be 11–12 weeks. The degradation process is accelerated by a low pH and acid-suppressing therapy (proton-pump inhibitors) is recommended to prolong stent integrity. A limited number of cohort studies have been reported with the ELLA-BDS for management of benign esophageal diseases such as benign esophageal strictures (BES), refractory benign esophageal stricture (RBES) [3,47,48,49,50,51,52,53,54,55,56,57,58,59,60,61,62,63,64,65,66,67,68,69,70,71,72,73,74,75] (Figure 6) and achalasia [61] (Table 1). Technical success, clinical responses and outcomes were different. Stent insertion was not a problem, however clinical success ranged from 0 to 100%, with a mean of 74.68**%**. Only a few studies contained ten or more patients. Repici *et al.* [57] reported dysphagia be completely alleviated in 43% of patients with RBES after EllA-BDS placement after a median follow-up of 53 weeks (range: 25–88 weeks); eight (26%) patients had recurrent dysphagia. Van Boeckel *et al.* [62] reported dysphagia be completely relieved in 33% of patients after a median of 166 days (range: 21–559 days); main complications take place in 22% of patients. Ibrahim *et al.* [58] included 20 patients management with an ELLA-BDS found that half patients required one or more additional stent insertion after a follow-up of six months. Moreover, Van Hooft *et al.* [63] concluded that an EllA-BDS insertion was an effective one-step management in 60% patients for an esophago gastric anastomotic stricture, obstruction recurred in 40% of patients and 30% of patients required endoscopic dilation after six months follow-up. Hair *et al.* [61] evaluated the efficacy and safety of sequential ELLA-BDS insertion in RBES patients. In sum, 59 stents were inserted in 28 patients. The mean dysphagia-free period was 90 days (range: 14–618 days) after initial stent placement. Clinical success (without dysphagia of ≥6 months) was acquired in 25% patients. The mean dysphagia-free period was 55 days (range: 25–700 days) with clinical success acquired in 15% of patients after a second BD stent insertion. The median dysphagia-free period was 106 days (range: 90–150 days) after the third BD stent insertion, but none of the patients were clinically dysphagia free. These studies concluded that a single ELLA-BDS placement is only temporarily effective in most patients. The major complications were vomiting and retrosternal pain. After one, two and three ELLA-BDSs insertion, the main complications take place in 29%, 8% and 28% of patients, respectively [66]. Sequential ELLA-BDS insertion can be an effective alternative for a severe RBES to avoid frequent serial dilations.

In contrast to the PLLA-BDS, the ELLA-BDS has shown some promising results. An initial case series with the ELLA-BDS for esophageal strictures [51,56,58,62,63] showed a low migration rate (0% to 22%) and an acceptable clinical success rate (33% to 60%). Current research has shown the uncovered ELLA-BDS allows stent embedding to the esophageal wall and significantly reduces the migration rate but can induce significant hyperplastic tissue reactions [52,53,55,61,63]. Nine studies systematic review of 157 patients with benign esophageal strictures showed the following results: 96% of technical success rate, 54.67% of clinical success rate, 13.98% of early stent migration rate, and 14.54% of tissue hyperplasia rate [48,49,50,62,64,70,71].

### 8.2. Malignant Stricture

Nowadays, neoadjuvant chemoradiotherapy improves long-term survival after esophageal surgery [76]. An interesting new concept is stent placement before neoadjuvant therapy in treat for resectable esophageal malignancy. It could be useful as a bridge to surgery during the neoadjuvant chemotherapy, oral solid intake improving nutritional status, nasoenteral or percutaneous feeding tubes not required. After termination of neoadjuvant therapy, esophagectomy is scheduled shortly, and stent-related complications can be averted. In several studies this approach has been evaluated using different types of stents and variety of neoadjuvant therapy [62,65,66,73,75,77,78,79,80,81,82]. Systematic review [66,68,71,75] of 57 patients with a malignant esophageal stricture obtained from four studies showed the following results: technical success rate of 96%, relief dysphagia rate of 79.5%, clinical success rate of 91%, early stent migration rate of 8.8%, and tissue hyperplasia rate of 14.5%. These studies appear effective in improving dysphagia and maintaining nutrition. Complications, though rare, were still occurring. Esophageal perforations and stent migration required urgent surgery. Stent migration has been reported to lead to small bowel perforation or obstruction.

### 8.3. Leaks or Perforations

Recently, modified ELLA-BDSs with a non-BD covering made of polyurethane were used in postoperative anastomotic leaks (*n* = 4) and benign esophageal perforations (*n* = 1) [83]. The initial technical success rate was 100%, clinical success rate was 80% and the stent migration rate was 60%. They considered that use of biodegradable material for covering the stents for the treatment of esophageal anastomotic leaks or perforations is technically feasible and safe. Nonaka *et al.* [84] designed a new biodegradable covered stent for the repair of emergent esophageal perforation in four pigs. The stent is composed of a 1:1 copolymer of PLA and PCL reinforced with PGA fibers. The stent was inserted inside the esophagus endoscopically to cover the perforation. It was observed at one week after the implantation but was wholly undetectable by two weeks. There was no stenosis or any kind of infection around the repaired wall. The BDS should be a useful strategy for managing esophageal wall injury such as esophageal ESD-related perforation.

At present, clinically available BD stent are the ELLA-BD stent (ELLA-CS, Hradec Kralove, Czech Republic), which is consists of polydioxanone (a surgical suture material) [55] and the PLLA-BD stent (Marui Textile Machinery, Osaka, Japan), which is composed of knitted PLLA monofilaments [48] (Table 2).

## 9. Biodegradable Drug-Eluting Stents (DES)

BDSs are known to be absorbed within 8–10 weeks, thus preventing long-term tissue reaction and stenosis. However, hyperplastic tissue reactions have emerged as a large issue. The ingrowth and overgrowth could cause delayed stent occlusion and restricted patency, resulting in a shortened patient survival. The covered design minimum impact on the hyperplastic tissue reaction is still an unsolved problem. Recently, drug-eluting stents (DES) have been recommended as a solution to the problem of reactive hyperplasia. Drug-eluting stents are expected to prolong stent patency by adding anti-hyperplasia or anti-tumor functions. Theoretically, a localized delivery of drugs such as paclitaxel or rapamycin from DES is a promising treatment method for preventing restenosis or inflammatory cell proliferation. As first-generation material of DES, biodegradable polymers represent the next technological modification, preliminary results are favorable in vascular system and clinical efficacy. Many experimental studies have been done in gastrointestinally with DES with animal models [2,85,86,87,88,89]. More studies on the clinical application of drug-eluting BD stents for human patients are need in the furture.

### Biodegradable Eluting Nanofiber-Covered Metal Stent

**Figure 7:**
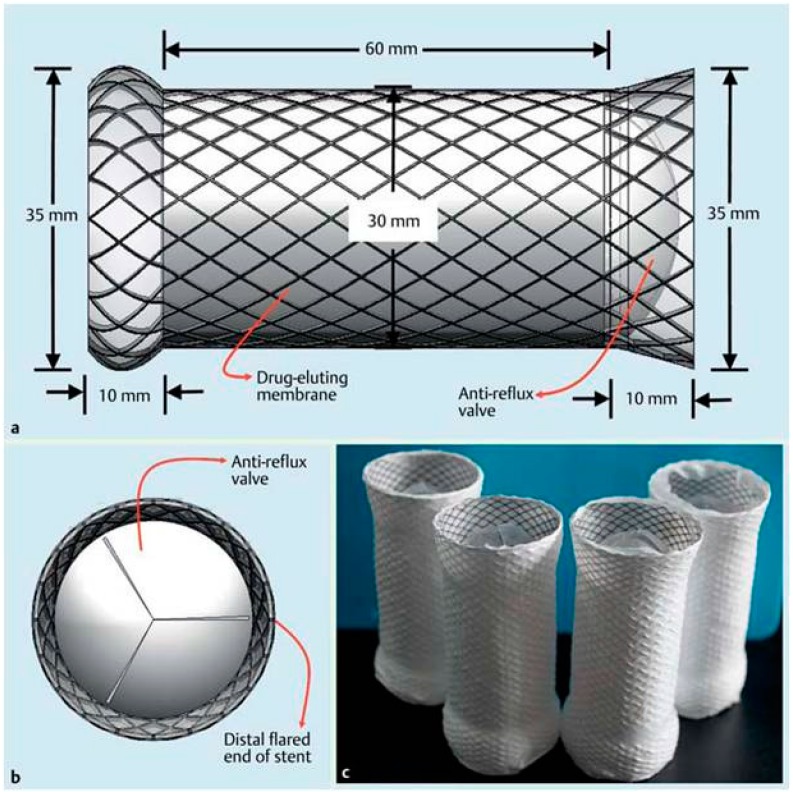
Diagrammatic and real representation of rapamycin-eluting stents: (**a**) the lateral view of the fully expanded bare cardia stent and its design parameters; (**b**) the antero-apical view of a schematic diagram of a stent with a trisected offset valve designed to prevent reflux and to allow smooth passage of food; and (**c**) overview of the fully expanded rapamycin-eluting stent [85]. Copyright permission from Thieme.

**Rapamycin-eluting**: Zhu *et al.* [85] evaluated a biodegradable rapamycin-eluting nano-fiber membrane-covered metal stent and to assess whether placement of this stent is followed by fewer fibroblast proliferation and tissue hyperplasia contrast to bare stents in experimental stricture in a dog model (Figure 7). They found that rapamycin-eluting stents were more effective than bare stents for significantly reduced fibroblast proliferation and tissue hyperplasia after stent implantation.

**Figure 8:**
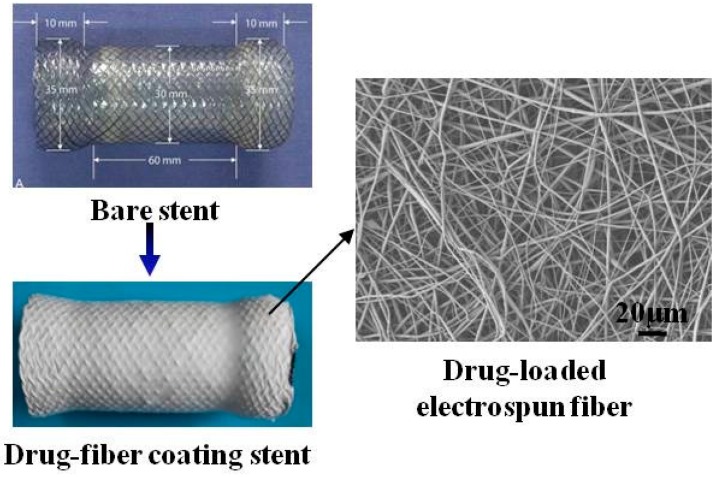
Fabrication stent coated with electrospun PCL fibrous membrane containing paclitaxel by fixing a bare stent on the rotor as the collecting setup for the treatment of benign cardia stricture [86]. Copyright permission from Elsevier.

**Paclitaxel-eluting**: Zhu *et al.* [86] evaluated a biodegradable paclitaxel-eluting nanofiber-covered metal stent for mangement of benign cardia stricture *in vitro* and *in vivo* (Figure 8). The DES of BD was more effective for management of benign cardia stricture than bare stents in a canine model. This study shown that a DES designed to limit fibrotic scarring is effective in a large animal model. This may become a safe and effective method for the management of benign cardia strictures in clinical practice.

**Biodegradable electrospun drug-fiber-coated stent**: Zhu *et al.* [87] designed and developed a new biodegradable electrospun drug-fiber-coated stent (DFCS). The electrospun paclitaxel/PCL fibers integrally covered the bare stent using the rotating collection method. Experimental studies have shown that this new designed biodegradable paclitaxel/PCL microfibrous membrane-covered stent was fabricated from blending electrospinning. The DFCS was a safe and effective method for the treatmentfor benign cardia stricture in dog model, and can avoided inflammation and scar formation. DFCSs may have great potential to treatment of stent-induced inflammation and scar formation in esophageal stricture therapy.

## 10. Complications of Biodegradable Esophageal Stents

Since BDSs caused more retrosternal pain, restenosis relate to hyperplasia and bleedings, complication rate of BDSs (28.6%) was twice higher than those of SEPS (14.3%) and FCSEMS (10.6%) [90]. The most frequent reported stent-related complication was thoracic pain. After BD stent insertion, severe thoracic pain occurred in 13.0% of patients. The retrosternal pain was caused by the radial force of the stent against the tight stenosis and was mainly reported within the first week after stent implantation [91,92,93,94,95,96]. However, through *in vitro* analysis of the radial and axial forces of 23 esophageal stent models, the BD stent had a relatively low radial force and high axial force [97]. Therefore, it is more likely that the rigid stent design of BDSs caused more spasm and pain due to peristalsis of the esophagus. 

**Figure 9:**
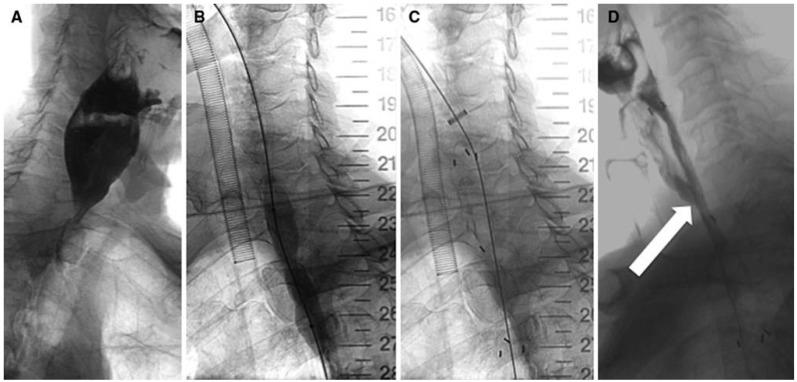
Patient with a high esophageal stricture due to recurrence of adenocarcinoma (**A**); contrast swallow showing the level of the stricture (**B**); a 14 mm × 60 mm balloon was used for predilation of the stricture (**C**); and a 25 mm × 60 mm biodegradable esophageal stent was inserted with the patient under general anaesthesia(**D**). Contrast swallow three days later shows stent proximal migration and the presence of a tracheoesophageal fistula (arrow) [71]. Copyright permission from Springer. Acute esophageal necrosis, a very rare condition of unknown aetiology, is defined as “a dark, pigmented state of the oesophagus (“black oesophagus”), with mucosal and submucosal necrosis at histology”. Tse A *et al.* [98] reported a case of acute esophageal necrosisafter biodegradable stent treatment for benign esophageal stenosis. Rejchrt *et al.* [99] reported three cases of black esophagus found on endoscopy, two patients died because their underlying disease. Other predisposing factors identified for this life-threatening condition include anticardiolipin antibody syndrome, herpes esophagitis, severe diabetic ketoacidosis, gastric volvulus and ruptured thoracic aortic aneurysm.

Hyperplasic tissue reactions occur in conjunction with stent degradation and the severity of the tissue response is another frequent complication. In one analysis, clinically relevant hyperplastic tissue growth was reported in 7.8% of BD stents [90]. Two case series studies also shown that reactive tissue formation is common after BD stent insertion [63,71] (Figure 9). Severe tissue hyperplasia caused recurrent dysphagia have been reported in some cases [51,57,61,64,69]. The reaction to the chemical processes of degradation resulting in tissue growth, which may also trigger bleedings of esophageal mucosa.

Other potential rare complications of BDS that have been addressed are biodegradable esophageal stents eroding into the tracheobronchial tree causing airway compromise [100], collapse of the biodegradable stent mesh inside the esophageal lumen [57,101], tracheoesophageal fistula [59] and a feeling of obstruction because food stuck in the BDS one months later [81].

## 11. Limitations of the Biodegradable Stents

There are several limitations of polymeric biodegradable stents [102]. BD stents are not as strong as metallic stents, which can cause early recoil after stent placement. Slightly lower radial force of BDS may impair their effect in dilation of tight stricture, water tight closure of leaks, and prevention of migration. After stent implantation, stent migration is a problem for BDSs. A major reason for this is located in the fact that the mechanical strength of the BD stent gradually suffered reduction of support force from polymer biodegradation, which potentially led to an invalid support against the stricture for a passage way. Therefore, further research the effect of *in vitro* polymer degradation on compressive strength of BD stent is need in the future. They are associated with a significant degree of local inflammation and the rate of bioabsorption is relatively slow, which may still result in restenosis. Furthermore, stents are radiolucent, which may influence accurate positioning. It is difficult to insert the stent smoothly and precisely without fluoroscopic guidance. A single polymer has a limited mechanical properties and a recoil rate of approximately 20%. BD stent require thick framework, but it influence on their profile and delivery capabilities, especially in small vessels [102]. The polymer stents require special storage conditions and have a shorter shelf life. Additionally, some polymer stents may also require a special delivery system.

## 12. Conclusions

BDSs have shown reduced migration rates owing to its uncovered design and early studies have reported excellent results compared to SEMS and SEPS. SEPSs and BDSs have their own special merits such as decreasing tissue hyperplasia and eliminating the need for stent removal. However, hyperplastic tissue reactions, thoracic pain and migration are the most frequent complication reported in the literature and have emerged as a main problem. When considering patients for insertion of a BDS，the severity of tissue response and the time to complete degradation of BDSs are important factors and are still not well acquaintance. The question of which type of BD stent should be recommend for the effective treatment of esophageal diseases has no clear answer. Therefore, the selection of type of BDSs for endoscopic management should be individualized. The experience of the endoscopist, patient and stricture characteristics, especially the location and cause of the stricture, should all be considered. To minimize tissue hyperplasia after BDSs placement, steroid injection or a drug-eluting BD stent may be an effective option. Currently, many experimental studies in DES have been done in the gastrointestinal region with animal models. In the future, more studies on the clinical application of drug-eluting BD stents for human patients are needed.

Recent advances in technology have improved patency and radial force of BD stent, reduced complications related to BD stent and resulting in an improved quality of life. However, the BDSs of esophagus continue to undergo design to overcome their limitations. The esophagus is an acidic environment that make the design and application of esophageal BD stent different from vascular and colorectal stents.Further technical refinements and studies to improve and demonstrate their efficacy are needed.

## Figures and Tables

**Table 1:** Result of biodegradable stents in esophageal strictures.

Study (Year)	Year	Stent Type	Study design	*n*	Indication	Follow-up	Relief dysphagia	Migration (%)	Hyperplasia (%)	Complication (%)	Clinical success (%)
Goldin *et al.* [47]	1996	PLLA Instent Improve Instent	Retrospective	3	BES	2–3 weeks	3(100)		3(100)	0	0
				2		2 months	2(100)	0	0	0	2(100)
Fry *et al.* [3]	1997	PLLA Instent	Case report	1	BES	6 weeks	1(100)	0	0	Proximal stent collapsed, removed	1(100)
Tanaka *et al.* [48]	2006	Tanaka-Marui Stent PLLA	Retrospective	2	BES	6 months	2(100)	0	0	0	2(100)
Satio *et al.* [49]	2007	Tanaka-Marui Stent PLLA	Retrospective	13	6 BES 7 RBES	7–24 months	13(100)	10(77) 10–21 days	0	0	13(100)
Satio *et al.* [50]	2008	Tanaka-Marui Stent PLLA	Retrospective	2	Cancer	7–24 months	2(100)	0	0	0	2(100)
Dhar *et al.* [51]	2009	ELLA	Prospective	4	RBES	4–17 weeks	4(100)	0	0	1(25)	4(100)
Vandenplas *et al.* [52]	2009	ELLA	Caes report	1	BES	10 months	1	0	0	Pain, vomit	1(100)
Orive-Calzada *et al.* [53]	2009	ELLA	Case report	1	BES	2 months	1(100)	0	1(100)	0	0
Bychkova *et al.* [54]	2009	ELLA	Case report	1	BES	6 months	0	0	0	0	1(100)
Stivaros *et al.* [55]	2010	ELLA	Retrospective	2	1RBES 1 RT	3–4 months	2(100)	2(100)	0	1(50) Pain(1)	2(100)
Viedma *et al.* [56]	2010	ELLA	Prospective	4	RBES	mean 24 weeks	4(100)	0	3(75)	0	3(75)
Repici *et al.* [57]	2010	ELLA	Prospective	21	RBES	25–88 weeks	9/20(45)	2(9.5) 7weeks	1(4.7)	4(18.7) Pain (3) Bleed (1)	9(43)
Ibrahim *et al.* [58]	2010	ELLA	Retrospective	20	RBES	mean 90 days	20(100)	0		12(60)	8(40)
Jung *et al.* [59]	2010	ELLA	Case report	1	RBES	4 months	1(100) 4 weeks	0	0	Tracheoesophageal fistula 4 weeks later	0
Güitrón-Cantú *et al.* [60]	2010	ELLA	Case report	1	RBES		1(100)	0	1		1(100)
Hair *et al.* [61]	2010	ELLA	Case repot	1	achalasia	8 weeks	1(100)	0	1(100)	stent collapse (Surgical resection)	0
Van Boeckel *et al.* [62]	2011	ELLA	Prospective	18	RBES	21–559 days	6(33)	4(22)	2(11)	4(22) Pain (2) Hematemesis (2)	6(33)
van Hooft *et al.* [63]	2011	ELLA	Prospective	10	RBES	6 months	6(60)	2(20)	2(20)	0	6(60)
Fischer *et al.* [64]	2012	ELLA	Retrospective	2	BES	12 months	1(50)	0	1(50)	0	1(50)
Canena *et al.* [65]	2012	ELLA	Retrospective	10	BES	11–21 months	10(100)	2(20)	3(30)	2(20) Pain (1) Hematemesis (1)	3(30)
Griffiths *et al.* [66]	2012	ELLA	Prospective	24	7 RBES + 17 cancer	6–8 months	17/22 (77) 12 weeks	2(8.3)	0	3(12.5) Bleed(1) vomiting(1) Hematemesis (1)	18(75)
Hirdes *et al.* [67]	2012	ELLA	Prospective	19	cancer	51–140 days	17(89)	0	2(11)	8(42) Hematemesis (1) pain (6)	17(89)
Hirdes *et al.* [68]	2012	ELLA	Prospective	28	RBES	14–618 days	15(54)	3(11)	0	11(40)	7(25)
Dumoulin *et al.* [69]	2012	ELLA	Case report	1	RBES	18 months	1(100) 4 month	0	1(100)	1(100) Pain 2 days	1(100)
Karakan *et al.* [70]	2013	ELLA	Retrospective	7	BES	36–102 weeks	7(100)	0	4(57)	0	7(100)
Krokidis *et al.* [71]	2013	ELLA	Retrospective	11	cancer	32–210 days	8(72) 32–201 days	3(27) 42–84 days	3(27) 111–201 days	1(9) aspiration 30 days	11(100)
Sanchez *et al.* [72]	2013	ELLA	Case report	1	RBES	20 months	1(100)	0	0	0	1(100)
Okata *et al.* [73]	2014	ELLA	Case report	1	RBES Four sessions	4 BDS the age of 5–8 years	1(100) 4–7 months	0	0	0	1(100)
Martín *et al.* [74]	2012	PLLA	Case report	1	Cancer ESD	7 months	1(100) 1 month	0	0	food stuck in the stents	1(100)
Van den Berg *et al.* [75]	2014	ELLA	Retrospective	10	cancer	93–166 days	8(80)	0	2(20)	Pain 6(60) 1–57 days	10(100)

**Table 2:** Currently Available Biodegradable Stents for Benign Esophageal Strictures.

	PLLA stent (Marui Textile Machinery, Japan)	ELLA stent (Hradec Kralove, Czech Republic)
Materials	Polyglycoside (knitted poly-l-lactic acid monofilaments)	Polydioxanone (a semicrystalline, degradable polymer)
Bioabsorption period	3–6 months	2–3 months
Length and diameter	Designed according to esophageal lesion	Size: 18, 20, 23, 25 mm Length: 60, 80, 100, 135 mm
Setting	Fitted over an endoscope	Delivery system
Other features	One end is reduced to a diameter of 5 mm by tying with silk sutures	Manual loading is needed

## Abbreviations

BDS, Biodegradable stent; BES, Benign esophageal strictures; CT, Computed tomography; DES, Drug eluting stent; DFCS, Drug-fiber-coated stent; EEM, Endoscopic esophageal mucosal-resection; EMR, Endoscopic mucosal resection; ES, Esophageal strictures; ESD, Endoscopic submucosal dissection; HBPPs, Hyperbranched polyphosphates; MLD, Minimum lumen diameter; MRI, Magnetic resonance imaging; PAGA, Polyacetyglutamic acid; PBTP, Polybutylene terephthalate; PCL, Polycaprolactone; PCLA, Poly (e-caprolactone-*co*-dl-lactide); PDLA, Poly-d-lactic acid; PDLLA, Poly(d,l)-lactic acid; PDX, Polydioxanone; PEO, Polyethylene oxide; PGA, Polyglycolic acid; PGLA, Polyglycolic acid/polylactic acid; PHBV, Polyhydroxylbutyratevalerate; PLA, Polylactic acid; PLLA, Poly-l-lactic acid; POE, Polyorthoesters; PAA-PEG, poly(acrylic acid)–poly(ethylene glycol); PLCLs, poly(lactide-ε-caprolactone); RBES, refractory benign esophageal strictures; SEMS, Self-expanding metal stents; SEPS, Self-expanding plastic stent; SIBS, styrene-*b*-isobutylene-*b*-styrene.
